# Transition of Mental Health Services from Institutional to Community-Based Care Abroad and Its Context for Slovenia—Advantages and Risks

**DOI:** 10.3390/ijerph22071066

**Published:** 2025-07-03

**Authors:** Katja Horvat Golob, Alenka Temeljotov Salaj, Brigita Novak Šarotar

**Affiliations:** 1 University Psychiatric Clinic Ljubljana, Chengdujska 45, 1000 Ljubljana, Slovenia; brigita.novak@mf.uni-lj.si; 2 Department of Civil and Environmental Engineering, Faculty of Engineering, Norwegian University of Science and Technology, NO-7491 Trondheim, Norway; alenka.temeljotov-salaj@ntnu.no; 3 Department of Psychiatry, Faculty of Medicine, University of Ljubljana, Vrazov trg 2, 1000 Ljubljana, Slovenia

**Keywords:** community-based care, deinstitutionalization, destigmatization, mental health, public health

## Abstract

Deinstitutionalization is a transition from psychiatric hospitals and other mental health institutions as the primary setting for treatment of individuals with chronic mental health disorders to a range of services, including psychiatric care, that support independent functioning of an individual within the community. The transition has been encouraged by guidelines from the European Expert Group and further specified in the Slovenian Resolution on the National Programme of Mental Health 2018–2028. This integrated systematic and narrative literature review includes 47 international articles from PubMed, along with information on Slovenian mental health legislation and its implementation, to provide insights into deinstitutionalization abroad and its relevance for Slovenia. Although the transition to community-based care is welcomed for promoting independence and respecting individuals’ wants, there are cases where institutional care remains necessary to ensure safety and treatment during the exacerbation of chronic mental health disorders. The quality of care and outcomes generally improve with community-based care. However, the closure of institutions can lead to many unintended consequences, such as the revolving door phenomenon and transinstitutionalization. Both the advantages of community-based care and the important roles of mental health hospitals and other institutions are emphasized.

## 1. Introduction

Deinstitutionalization or the transition from institutional to community-based care as the primary setting of treatment and care of patients with mental health issues originated from social movements in the beginning of the last century [1]. Institutional care is generally used as a term for psychiatric hospitals and social welfare institutions [2]. Psychiatric hospitals are institutions that provide treatment to patients with acute mental disorders and acute exacerbations of chronic mental illnesses. Most patients need further psychiatric care after completing acute treatment and their discharge from hospitals, which can be done in an outpatient setting. However, some individuals, due to the chronic nature of their illness and resulting functional impairment, need further care and protection, which can be provided through social welfare institutions [3]. This article examines the impact of deinstitutionalization abroad and contextualizes the ongoing transition of mental health care in Slovenia from an integrated biopsychosocial perspective. It welcomes its influence on patients’ well-being and destigmatization, while warning against complete closure of institutional care.

Stigma toward mental health disorders has influenced decision-making regarding the care of psychiatric patients in the past and is still prevalent today. It is associated with both people who suffer from mental health illness and the institutions that treat and care for them. It negatively influences access to care and leads to delay in treatment, increased morbidity, and diminished quality of life of individuals with mental health disorders [4]. The built environment has been known to influence mental health and well-being [5]. Stigma contributed to inadequate financial support and poor facility management of these institutions in the past. Mental health facilities are criticized for poor design, which can worsen patients’ well-being and potentially promote problematic behaviors [6]. Prolonged institutional care can even develop into institutionalism, i.e., a pattern of passive and dependent behavior, which can delay the discharge process or complicate reintegration into the community [7]. Conversely, a well-designed built environment can reduce stress levels, and the intentional architecture and design of mental health institutions can contribute to positive health outcomes [8,9]. In the past, due to stigma, psychiatric hospitals used to be placed in secluded asylums [10]. In Slovenia, these were established in buildings originally designed for other purposes, such as old and run-down castles that are no longer in use [10].

At the beginning of the last century a new social movement, deinstitutionalization, started to spread across Europe, opposing institutional care and criticizing it for isolating residents from their communities, for denying patients control over decisions that affect them, and for institutional requirements taking precedence over individual needs [2]. The initiative promoted not only the closure of institutions but also the development of a range of community-based services. In 1960, this social movement began to influence Slovenia’s approach to mental health services as well. Various civil initiatives advocated both for the reintegration of mental health institution residents to the community and for structural and organizational reforms within the facilities [11,12]. The success of this movement has varied across Europe. A prominent example of the profound shift toward deinstitutionalization occurred in our neighboring country, Italy. Psychiatric institutions were closed, and psychiatric wards were integrated within general hospitals with variable results [10,13]. Slovenia’s psychiatric institutions remained functional, but some were relocated closer to the urban areas, and all were renovated.

Common European Guidelines on the Transition from Institutional to Community-Based Care were published by the European Expert Group on the Transition from Institutional to Community-Based Care in 2012 to ensure deinstitutionalization within the European Union [2]. These guidelines involve measures to prevent the need for institutional care, as well as measures to reintegrate individuals who have already resided in institutional care. They argue that institutions were once seen as the best way of caring for populations with different needs but ultimately attributed to their poorer quality of life and social exclusion. Their work is based on the United Nations Convention on the Rights of Persons with Disabilities, which recognizes the right to live independently in the community, and the European Convention on Human Rights, which guarantees the right to private and family life regardless of the nature of the impairment, as well as involvement in all decisions regarding one own’s life. Interference with the latter must be necessary and proportionate. This is further recognized by the World Health Organization, which advocates for a shift to community-based care, arguing that it leads to better outcomes in quality of life, greater cost-efficiency, and stronger respect of human rights. Supporters of deinstitutionalization warn against medicalization and the medical model of disability, which assumes that the disability is caused by a person’s impairment. They argue that the medical model focuses solely on treating individuals’ impairments and promotes segregation. In contrast, they support a social model, which attributes the disability to environmental barriers and focuses on inclusion. The European Expert Group emphasizes that adaptive behavior, such as self-care, communication, academic, social, and community skills, improves, and challenging behavior reduces with the transition to community. Community or independent living should not imply full self-sufficiency but rather the freedom to choose where to live, whom to live with, and how to organize one’s own daily life. The accessibility of the built environment, including transport, technical needs, information, and access to personal assistance and community-based services, plays an important role in this transition [2].

Psychiatric hospitals, as well as social welfare institutions in Slovenia, are still operating but have become closely regulated with legal framework and regulations. An increasing number of studies support the finding that the environment of institutional care influences patients’ recovery [14]. Within the existing structure, changes have been made to improve comfort while ensuring treatment and maintaining security. In Slovenia, facilities and hospital admissions are regulated by the Mental Health Act, which closely monitors the admissions process and special safeguarding measures to ensure respect for personal dignity, human rights, and fundamental freedoms, while also promoting individualized care [3]. Admission to closed wards is usually reserved for acute exacerbations of mental health disorders, with the intention of securing a person’s own safety and the safety of others while initiating treatment. A higher degree of supervision in closed wards is achieved through physically enclosed spaces, an increased number of medical personnel, and special safeguarding measures, while following strict protocols. Closed wards must accommodate no more than two patients per room, provide sufficient space for daily activities, ensure at least two hours of outdoor time per day, and include designated smoking areas. Although patients generally consent to the treatment plan and placement in a closed ward, involuntary hospitalization or placement is possible in an emergency and can be enforced by a court order. Involuntary admission can only be carried out if the less restrictive measures, such as admission to an open ward, outpatient treatment, or supervised treatment, are deemed inappropriate [3].

Social welfare institutions provide continuous special protection and accommodation for individuals that no longer require hospital treatment but still need special care. The law establishes a special form of outpatient treatment called supervised treatment. It is reserved for individuals with severe, chronic mental illness, who have a history of autoaggressive and heteroaggressive behavior, and it is enforced by a court order. This ensures adherence to treatment and helps prevent the recurrence of such behavior, while aiming to avoid institutional placement [3].

The Resolution on the National Programme of Mental Health for the years 2018 to 2028 is the first strategic document on mental health in Slovenia [15]. It assesses the current state of mental well-being in the population and proposes a plan to integrate various community-based actions. It identifies different population groups, each with their specific strategic goals, as well as planned actions to address the major public mental health problems in Slovenia, namely, suicidality and alcoholism. The assessment of institutional mental health care is based on the number of hospitalizations in psychiatric hospitals, which, at the time of the publication, were reportedly below average, while their duration was said to be slightly above the European Union average [16]. Outpatient treatment was relatively sparse, and psychiatric hospitals played a central role in mental health care in Slovenia, a country with approximately two million inhabitants. The resolution substantiates these statements with the following data. In 2016, a total of 4000 residents with chronic mental health disorders and other disabilities under the age of 65 lived in social welfare institutions. Most residents resided in larger departments, while only a few stayed in residential units. The number of institutionalized individuals in 2016 was among the highest in Europe. This emphasis on institutional care is further reflected in data showing that 80% of psychiatrists worked in psychiatric hospitals and other institutional settings [15].

The content of the national resolution is further summarized in the following paragraphs, along with its priorities and a more detailed examination of the action plan. One of the priorities of the national program is the transition to a community-based approach. The document emphasizes individuals’ and relatives’ choice and contributions in planning, implementation, and supervision of different mental health services. The services are designed as a multidisciplinary collaboration, incorporating primary healthcare with health promotion centers, psychiatric hospitals with emergency services, institutional care, mental health centers, community-based treatment coordinators, user-run associations, counseling services, various programs, and social work centers. According to the resolution, 25 new adult mental health centers are to be established, strategically distributed across all regions of Slovenia. These centers should provide triage, crisis intervention, early diagnostics, and treatment of mental health disorders, as well as psychotherapy for individuals, couples, and families. One of their key envisioned tasks is providing acute psychiatric treatment in a home environment, along with intensive monitoring and management of unstable mental health conditions. As such, these centers play a role in preventing the need for hospitalization and institutionalization. When necessary, the resolution predicts their collaboration with other organizations, e.g., referring patients to hospitals and subsequently promoting their return to the community [15].

With the establishment of accessible, comprehensive, and quality community services, the resolution anticipated a 40% reduction in residential capacity in mental health care institutions with planned relocation of their residents to the community. The action plan includes building dedicated residential units. To facilitate the integration of residents who have lived in mental health institutions for years, it provides services and measures to support housing, education, employment, and social activities. Residential groups’ programs offer varying levels of support, allowing individuals to transition between them as their needs change. These programs support individuals starting or returning to school or university and assist with employment. Day care centers offer socialization and activation. Additional services are designed for patients with comorbidity with addiction disorder. For those who need special protection and therefore reside in institutions, the document emphasizes the protection of human rights and dignity within facilities. This is achieved through adequate staffing levels, proper training, and the structural design of the facilities [15].

Researchers and clinicians abroad have expressed their concern in the complete transition to community care. Some countries report a surge of criminality and incarceration of people with severe mental health disorders after the closure of psychiatric hospitals [17,18,19,20,21]. Moreover, the disorders of the imprisoned patients tend to present even more severely than those treated in mental health hospitals [22]. The patients may not be necessarily violent themselves but are more vulnerable to being victims of violent crime in the community [23]. Some studies observe that deinstitutionalization contributes to a worsening of physical health and even to an increased mortality, from both cardiovascular causes and unnatural deaths, including from suicide [20,24].

## 2. Materials and Methods

**Figure 1:**
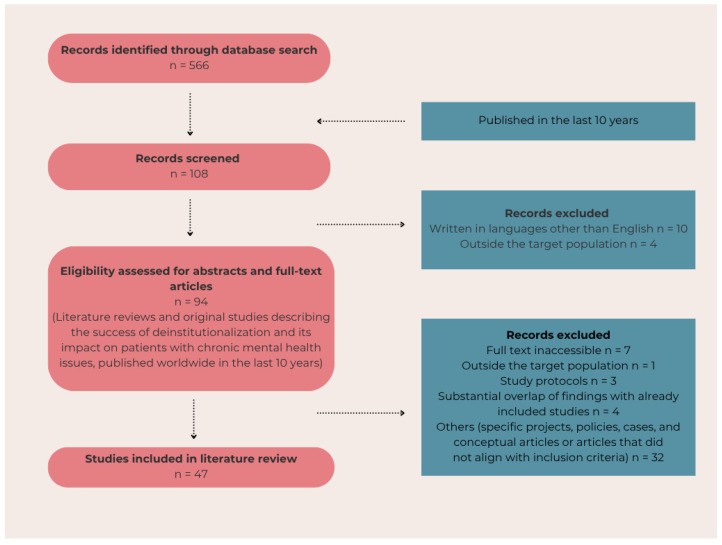
The flow diagram of the literature review.

The article integrates a systematic and narrative literature review. The literature review was conducted with the aim of determining whether the ongoing deinstitutionalization process in Slovenia has been studied from a biopsychosocial perspective. The electronic databases PubMed, PubMed Central, and ScienceDirect were used. As only a few Slovenian articles were found on these databases, we broadened our search to worldwide articles on the same topic. Different combinations of keywords were searched, with the most relevant results found on PubMed with the keywords ((deinstitutionalization) OR (deinstitutionalized)) AND ((community care) OR (psychiatric hospital)) AND ((effect) OR (outcome) OR (result)) NOT children NOT elderly, which resulted in 108 articles. The inclusion criteria consisted of literature reviews and original studies describing the success of the transition to community care and its impact on patients with chronic mental health issues, published worldwide in the last 10 years. Exclusion criteria, on the other hand, included inaccessible articles; articles written in languages other than English; study protocols without results; articles with substantial overlap of findings with those already included; studies focusing on the deinstitutionalization of children or older adults; studies addressing outcomes of specific projects, policies, cases, and conceptual paper; and articles that did not align with inclusion criteria. One Slovenian article related to deinstitutionalization was found with this keyword search but was not included, since it focused on older adult and long-term care nursing homes, which exceeds the scope of this review. The articles were screened for their relevance based on abstracts and full-text reviews. The data from the remaining 47 articles were extracted, including their authors and year, title, study design, and key findings. The flow diagram of the literature review and the findings are presented in Figure 1 and Table 1, respectively. These were synthesized and analyzed for methodological approaches, similarities, and differences in perspectives. In addition, a narrative review regarding legislation related to mental health care and transition to community care in Slovenia and European Union was conducted, including the Slovenian Mental Health Act, the Resolution on the National Programme of Mental Health 2018–2028, the Resolution on the National Social Welfare Program for the Period of 2022 to 2030, and the Common European Guidelines on the Transition from Institutional to Community-based Care, as well as the results of the National Mental Health Program, MIRA Program, and Comprehensive Evaluation of the Implementation of the Action Plan 2022–2023.

## 3. Results

### 3.1. Literature Review

Deinstitutionalization process and community services vary across countries and are therefore challenging for interpretation; moreover, the results are often contradictory. Most countries observe reductions in available beds in psychiatric hospitals and show benefits of community-based care [27,33,37,54,57,66]. On the other hand, while the total number of beds was reduced, there was an increase in the number of psychiatric beds in general hospitals and forensic hospitals and short-stay beds in psychiatric hospitals [19,68]. Evidence that community care improves the quality of care and health outcomes has been shown in fifteen systematic reviews and a retrospective control study [36,41]. A systematic review found that most patients can be successfully discharged from long-stay hospitals to community settings without any clinical deterioration [43]. Transition to community care is beneficial toward patients, regardless of the country’s current level of deinstitutionalization [46].

Some authors describe a link between deinstitutionalization and poorly realized community care that leads to homelessness, crime, and imprisonment, a phenomenon referred to as transinstitutionalization [25,26,27,32]. One review reports an uptick in the suicide rate, while a cohort study indicates that homelessness, imprisonment, and suicide occur only sporadically [26,29,30]. On the other hand, empirical studies have found increased crime rates in Latin America and Korea [40,70]. A comparative cross-sectional control study documented a decline in the suicide rate in Finland [51]. The revolving door effect has been observed, with frequent readmissions to hospitals or patients rotating between shelters, jails, and hospitals [28]. This phenomenon is even more pronounced with rapid discharges and fixed-fee admissions [31,59,62]. A short length of stay below a critical threshold might increase morbidity and mortality [65].

The fewer beds or hospitals that are left are increasingly occupied with patients that are more challenging to treat [33]. Another effect of deinstitutionalization, called boarding, was identified, where patients seek help for mental health issues in insufficiently equipped emergency departments [39]. Deinstitutionalization leads to a change of structure in patients that seek urgent help, with a growing proportion of patients with substance use disorders, personality disorders, trauma-related disorders, and neurodevelopmental disorders that need acute additional protection. A decrease in other diseases, such as depressive disorder, was noted, which seem to be managed well in the community [61]. Furthermore, a notable rise in the use of restraints has been observed, likely due to higher-acuity patients. Great emphasis should be put on the de-escalation and provision of single-occupancy rooms to decrease incidents [61].

A community treatment order is a community-based alternative to forensic psychiatric institutions, which treat patients with mental illness who are involved in legal matters. It helps omit hospitalization, while also enabling rapid admission in cases of mental health deterioration [31,52]. While community care can be a good choice of treatment for many people with severe mental illness, some patients, particularly those with a history of repeated violence, require long-term psychiatric care facilities [28,32].

Deinstitutionalized people often return home, increasing the burden on family members, which has been described by qualitative studies, as well as systematic literature reviews. Family has to cope with the symptoms of the patients, provide care and support, and in cases of exacerbation of health issues may need to decide to involve the police [32,49,65]. Moreover, family members are the most likely targets of violent incidents [45]. The return to the home environment varies, and countries with lower family support tend to observe higher transinstitutionalization [51]. One study reported that due to the distress experienced by family members following discharge without additional support, many expressed a desire for the patient to return to an institution [55].

Countries have reported uneven redistribution of resources and emphasize that the policies should be tailored to the income of the country [29,33,34,58,60,67]. The deinstitutionalization efforts are often influenced by the current broader political situation [42,47,69]. In general, deinstitutionalization in Europe is reasonably successful based on a cross-sectional study and The Quality Indicator for Rehabilitative Care [53]. Most European countries provide both institutional and community-based mental health care, and their transition between the two is not strongly correlated with the number of psychiatric beds per capita [64]. The balanced care model has been proposed to integrate both hospital and community-based services, to provide access for people that need continuity of follow-up and acute treatment during mental health crises [34,44]. More data is needed to determine the minimum number of psychiatric beds per capita required for quality care and patients’ safety [65].

Transition to community care is sometimes unsuccessful, despite multi-professional involvement, and can lead to transinstitutionalization instead of reintegration to the home environment [35]. Community services might be underused, and some deinstitutionalized patients must return to a fully staffed institution. Although deinstitutionalization is associated with higher quality of treatment, a cross-sectional study observed that it was not significantly related to patients’ quality of life or their experiences of care [25]. Instead, the key factors related to the quality of life include symptom severity, low level of social skills, limited self-care, and poor education [63].

Italy, with its Mental Health Reform in 1978 (Law 180), was the first country with a radical transition from psychiatric hospitals to community care. One narrative review showed that neither crime rates nor suicide rates increased. The identified challenges included regional inequality, insufficient human resources, and a lack of scientific research on the process [38]. New legislation addressed the need for forensic psychiatry, introducing community-based structures yielding mixed results [48,50]. The opening of community care centers for forensic patients demonstrated improvement in mental health after the discharge, a reduced need for high-control environments, and a high rate of workplace integration. Reduction in health care costs and positive feedback among mental health workers were reported. However, crucially, the study showed high mortality from the initial discharges (18.2% of the sample of 55 people) and early death (average age of 49 years old) [56].

### 3.2. Current Mental Health Services in Slovenia

In 2025, there are 19 community-based mental health care centers with interdisciplinary teams, dedicated to the adult population. Additionally, 917 providers are officially registered in healthcare and social services for mental health support, offering free services to users. The information about these institutions is listed on the webpage Mira—National Programme of Mental Health with a search tool that displays programs based on the location or the nature of mental health issues. These providers include psychiatric care, temporary accommodations, consultations, educational programs, youth centers, advocacy, social skills training, humanitarian aid, and various other services [71].

Slovenia has six psychiatric hospitals located across different regions and five social welfare institutions with their smaller, dislocated residential communities. These institutions employ both healthcare workers and other professionals, e.g., social workers. The residents have regular access to family medicine specialists, psychiatrists, and dentists. The number of employees is regulated by law, but they are not necessarily present throughout the entire day. In contrast, community services called residential communities offer a more independent form of housing support. These communities are smaller, accommodate fewer people, and are staffed primarily by non-medical personnel who are generally present only on weekday mornings. The strictness of monitoring medication adherence is usually lower than that of traditional institutions. Their structure is prescribed by The Resolution on the National Social Welfare Program for the Period of 2022 to 2030 and accommodates up to 24 users in each unit [72].

The extent of the effort and appreciation for community-based approach in Slovenia is best described by the following data. The statistics from 2022 show 390 residents of residential groups, 2370 users of day care centers, 1750 users of counseling, 21,587 users of telephone counseling programs, and 617 users of advocacy for mental health. Three different organizations offer helpline counseling service [71]. The Centre for psychological counseling Posvet, located in 18 different cities, conducted 6875 individual counseling sessions and helped 3670 times over the helpline in 2024 [73]. The most prominent examples of deinstitutionalization in Slovenia are the organizations Center for Training, Work and Protection Črna na Koroškem and Dom na Krasu Dutovlje, which are both active in returning residents to the community. The facilities reintegrated 96 and 70 of the former institutional residents [74].

## 4. Discussion

The process of deinstitutionalization in Slovenia is well documented in the social science literature. These studies emphasize the importance of patient autonomy and trace the historical development of the movement, while also critiquing mental health professionals for their cautious and at times reluctant acceptance of social change [12]. The significance of community-based mental health services in Slovenia has also been highlighted by clinicians [75]. These studies and perspectives are valuable as they initiate necessary dialogue and promote improvements in the quality, safety, and efficacy of treatment. On the other hand, little has been said so far about the risks of this transition and dangers of the complete closure of institutional care in Slovenia. The biopsychosocial perspective is under-represented and under-researched, especially following the adoption of the Resolution on the National Programme of Mental Health, which advocated for a further and accelerated shift toward community care.

Community-based mental health services aim to reduce stigma and improve patient outcomes. These changes promote autonomy and, according to the World Health Organization, enhance the well-being and life satisfaction of people with mental health disorders [76]. As patients’ well-being is inherently connected also to the remission of their illness, Slovenian law recognizes the need to provide prompt and appropriate treatment, even when it may override an individual’s sense of autonomy, in order to ensure their safety and rapid recovery [3,63,77].

Some hospitalizations are due to truly acute conditions, i.e., suicidal ideation during an acute stress reaction, which require immediate treatment and quick resolution. On the other hand, some mental health disorders are chronic, progressive disorders that require continuous treatment. An example of the latter is schizophrenia, one of the fifteen leading causes of disability worldwide [78]. Initially, with proper treatment, hallucination and delusions are only transitory, and the patient may gain insight or come to understand that these experiences are not grounded in reality. As the illness progresses, delusions may become more grandiose and persistent. Perhaps even more damaging to the daily function are other symptoms, such as avolition, social withdrawal, and progressive cognitive impairment. The progression of the disease results in important social and occupational impairment [79].

As clinicians working in psychiatric hospitals, we encounter individuals with various psychiatric disorders who have exhibited violent intentions, toward either themselves or others [80]. In rare cases, this behavior is chronic and unpredictable and can lead to prolonged institutionalization to ensure safety and protection [81,82]. Globally, there is a recognized need for a balanced care model that integrates both community-based and institutional care [34,65].

Global studies highlight potential negative consequences and warn against the complete closure of institutional care. Some studies observe a higher rate of suicide, while others report a surge of criminality and incarceration of people with severe mental health disorders after the closure of psychiatric hospitals [26,40]. This so-called Penrose hypothesis has been observed in Slovenia between 1990 and 2018; however, the effects of even more pronounced transition to community-based care after 2018 have not been studied [83]. Although our systematic literature review challenges these negative claims and includes studies that, in contrast, present evidence of a reduced suicide rate, we urge caution in drawing conclusions as they involve serious consequences. It has been proposed that increases in suicide rates and all-cause mortality may occur if the number of available beds falls below a critical threshold [65]. Further data and studies on the topic are needed.

Surprisingly, the quality of life of the patient might not necessarily increase with deinstitutionalization, whereas family members often experience a heightened burden [84]. Whether the transition resulted in actual inclusion in the broader community is unclear [20,63]. The effects such as transinstitutionalization, boarding, and revolving doors are concerning. Italy reports great success in their deinstitutionalization efforts of forensic patients but simultaneously records high mortality and early death [56].

To our knowledge, this is the first review to integrate Slovenian transition to community care with outcomes of deinstitutionalization abroad, which emphasizes both advantages and risks. Slovenia has made significant and comprehensive steps toward a community-based approach. We believe this to be beneficial for most patients. Despite all the support that community offers, it sometimes still proves to be insufficient. As clinicians working in psychiatric hospitals and mental health institutions, we can attest to several cases where deinstitutionalization has led to unequivocally negative outcomes. This is the case with patients who have severe mental health disorders and require additional protection. Due to reductions and limited availability of institutional capacities, transfer from hospitals to welfare institutions is not possible. These individuals remain hospitalized for months, sometimes even years, in either closed or open wards while waiting for a placement in a social welfare institution. This uncertain situation is unjust for the individual and places additional strain on the overburdened health care system. Although community services are designed to manage acute episodes and prevent admissions and institutional placements, hospitalizations are frequent, and social welfare institutions are in high demand.

Instead of focusing solely on the reduction in institutional capacities, we suggest ongoing, evidence-based efforts toward user-friendly facilities with therapeutic design; strong emphasis on training of mental health professionals; public education to combat stigmatization; and, nonetheless, sufficient funding to make this possible. Further scientific documentation and research are essential to inform future policies and directions.

### Limitations

Despite our best efforts, we found very little data on the transition to community care in Slovenia following the implementation of the National Resolution on Mental Health. As such, our conclusions on the current success of deinstitutionalization in our country are limited. Moreover, we describe our clinical experience that resulted from bed reductions in hospitals and social welfare institutions in the discussion but are unable to support it with objective findings. Further empirical studies are necessary to document and evaluate the outcomes of the transition to community care in Slovenia, including its effect on quality of life, disease remission, mortality, caregiver burden, and criminality and its financial implications.

## 5. Conclusions

Slovenia has taken active steps toward deinstitutionalization and a community-based approach, guided by the Common European Guidelines on the Transition from Institutional to Community-based Care, the Mental Health Act, and the Resolution on the National Programme of Mental Health 2018–2028. These efforts brought positive changes to ensure patients’ well–being and expanded the availability of mental health services to people with mental health issues. However, a complete transition and closure of mental health institutions carries risks. Psychiatric hospitals play a fundamental role in ensuring safety, diagnostics, and treatment during acute phases of psychiatric illness, while social welfare facilities provide support for individuals who require ongoing care due to the chronic nature of their conditions. Instead of moving toward complete closure of mental institutions, we propose continued efforts to ensure the comfort and dignity of patients while providing care and treatment during their most vulnerable moments. The optimal approach to mental health care is the integration of institutional and community-based care. In addition to strengthening versatile community programs, we propose increased funding and support of mental health institutions, along with expanded capacity, to ensure better care of our patients.

## Figures and Tables

**Table 1:** The literature review.

N.	Title	Authors	Journal	Study Design	The Main Findings
1	A brief history of the criminalization of mental illness.	Dvoskin et al., 2020 [25]	*CNS Spectrums*	Narrative review.	Although mental illness accounts for a small percentage of violent crime, deinstitutionalization and inadequately funded community care have contributed to homelessness and imprisonment. To prevent inappropriate incarceration, police officers and first responders need to be trained in recognizing mental disorders or emotional crises. Additionally, sufficient hospital beds must be made available for short-term hospitalization and crisis stabilization. Mental health services should also be accessible within jails and prisons.
2	The fiftieth anniversary of the article that shook up psychiatry.	Rybakowski J., 2023 [26]	*Psychiatria Polska*	Narrative review.	Poland faces a lack of available psychiatric beds after implementing the National Program of Mental Health 2011–2015. A significant correlation has been observed between the reduction in psychiatric beds and an increase in suicides. As a result, patients are often left with only two options: homelessness or imprisonment. In 2022 in the USA, a phenomenon transinstitutionalization was noted, with ten times more psychiatric patients placed in prisons than in psychiatric hospitals.
3	Organization of Community Psychiatric Services in Finland.	Korkeila J., 2021 [27]	*Consortium Psychiatricum*	Narrative review.	Mental health services in Finland are mostly outpatient-oriented, with the number of available beds reduced to just one fifth of what was available four decades ago. Deinstitutionalization has not resulted in a rise in the mortality rate or homelessness of individuals with mental health issues. As in Denmark, patients with schizophrenia, who are more challenging to treat, are increasingly referred to state mental hospitals, which are the only psychiatric facilities where the number of wards and beds has increased.
4	Through the Cracks: The Disposition of Patients with Schizophrenia Spectrum Disorders in the Post-Asylum Era.	Tillman, et al., 2022 [28]	*HCA Healthcare Journal of Medicine*	Narrative review.	Improving access to affordable housing for patients discharged from psychiatric facilities could help disrupt the “revolving door” cycle between shelters, jails, and hospitals. While community care can be a better treatment option for many individuals with psychotic illness, a significant portion of these patients would still benefit most from long-term psychiatric care facilities.
5	Deinstitutionalization does not increase imprisonment or homelessness.	Taylor Salisbury T., Thornicroft G., 2016 [29]	*The British Journal of Psychiatry*	Narrative review.	Most studies on deinstitutionalization report no cases of homelessness, incarceration, or suicide following discharge from the hospital. As outcomes are closely connected to the level of funding of community care services, it is essential to define an appropriate balance of hospital and community-based care across low-, middle-, and high-income countries.
6	Deinstitutionalized patients, homelessness and imprisonment: Systematic review.	Winkler, et al., 2018 [30]	*The British Journal of Psychiatry*	Systematic review.	Cohort studies that followed deinstitutionalized patients showed that they benefited from the transfer to the community care, with serious behavioral issues, such as homelessness, imprisonment, or suicide, occurring only sporadically.
7	Hospital Utilization Outcomes Following Assignment to Outpatient Commitment.	Segal S.P., 2022 [31]	*Administration and Policy in Mental Health and Mental Health Services Research*	Systematic literature review.	Outpatient civil commitment, also known as a community treatment order, requires noncompliant patients to receive necessary treatment, addressing threats to health and safety. This treatment is community-based and a less restrictive alternative to psychiatric hospitalization. It helps limit hospitalization, while also ensuring that patients in mental health crises are referred to necessary treatment in a hospital. Additionally, rapid discharges are associated with a higher likelihood of readmission, particularly in hospitals operating under fixed-fee per admission models.
8	Deinstitutionalization and other factors in the criminalization of persons with serious mental illness and how it is being addressed.	Lamb H.R., Weinberger L.E., 2019 [32]	*CNS Spectrums*	Narrative review.	Deinstitutionalization is one of the contributing factors to incarceration of individuals with mental illness. Family members are often left to manage the patient’s symptoms while also coping with their own emotional responses and deciding whether to involve the police, particularly in situations involving violence. While community care can be an appropriate treatment option for individuals with a history of incarceration, those with severe mental health disorders and a history of violence require specialized, highly structured, and adequately secured clinics.
9	Advances and challenges of the Back Home Program as a deinstitutionalization strategy: An integrative review.	De Pádua Lima H., et al., 2022 [33]	*Ciencia & Saude Coletiva*	Integrative literature review.	Between 2002 and 2015, more than 58% of psychiatric hospital beds in Brazil were closed, accompanied by the implementation of various substitute services. However, the availability of community care is unevenly distributed across the country and is heavily influenced by the current political climate.
10	Community mental health care worldwide: Current status and further developments.	Thornicroft G., et al., 2016 [34]	*World Psychiatry*	Narrative review.	The balanced care model is proposed as a conceptual framework for providing both hospital and community-based services, to ensure access to care during mental health crises and maintain continuity of follow-up. This model must be adapted to the economic level of each country.
11	Deinstitutionalization and network of mental health services: A new scene in health care.	Medeiros Lima A., et al., 2020 [35]	*Revista Brasileira de Enfermagem*	Qualitative study.	The process of deinstitutionalization at the hospital studied in Brazil took longer than initially estimated. It required multisectoral involvement, including managers, healthcare professionals, and service users. Most patients were referred to therapeutic or transinstitutionalized residences, while only a small portion returned to live with their families.
12	Improving Care for Deinstitutionalized People with Mental Disorders: Experiences of the Use of Knowledge Translation Tools.	Fulone I., et al., 2021 [36]	*Frontiers in Psychiatry*	Systematic literature review.	The available evidence from fifteen systematic reviews identifies six types of strategies (psychoeducation, anti-stigma programs, intensive case management, community mental health teams, assisted living, and interventions for acute psychiatric episodes) that can improve the care for deinstitutionalized people with mental disorders and their health outcomes. More rigorous systematic reviews are needed to support evidence-based policymaking.
13	Deinstitutionalization and autonomy: Outcomes from a Brazilian mental health policy.	Andrade da Silva G., et al., 2022 [37]	*Ciencia & Saude Coletiva*	Multi-methodological research.	Research supports the effectiveness of diverse deinstitutionalization strategies in Brazil, but their operationalization could be further improved.
14	Mental health care in Italy: Basaglia’s ashes in the wind of the crisis of the last decade.	Carta M.G., et al., 2020 [38]	*International Journal of Social Psychiatry*	Narrative review.	The closure of psychiatric hospitals in Italy did not lead to an increase in crime; in fact, suicide rates have decreased since their closure. However, there are a few challenges in Italy’s community care, including a lack of scientific documentation regarding the deinstitutionalization process and insufficient funding. Furthermore, there are regional disparities in care, with a clear risk emerging from the imbalance between limited human resources and the need for mandatory medical treatment.
15	Boarding of Mentally Ill Patients in Emergency Departments: American Psychiatric Association Resource Document.	Nordstrom K., et al., 2019 [39]	*The Western Journal of Emergency Medicine*	Narrative review.	The article identifies two primary motivations for deinstitutionalization. One is scientific and patient-centered, driven by improved differentiation between acute and subacute risk, innovations in outpatient and crisis care, advancements of psychopharmacology, and growing recognition of the negative effects of coercive hospitalization. The other motivation is budgetary in nature. Due to the lack of appropriate services, the phenomenon of boarding has emerged, wherein patients seek care in medical emergency departments, settings not designed for the needs of individuals with mental illness, and wait there for hours or days before receiving appropriate treatment. The article suggests recommendations to improve care, both within medical emergency departments and at the broader community level.
16	Psychiatric beds and prison populations in 17 Latin American countries between 1991 and 2017: Rates, trends and an inverse relationship between the two indicators.	Siebenförcher M., et al., 2022 [40]	*Psychological Medicine*	Longitudinal ecological study.	The decrease in the availability of psychiatric beds in Latin America has been associated with an increase in incarceration rates. Policies establishing minimum standards for hospital bed availability and promoting the further development of community-based services should be implemented.
17	Aging of severely mentally ill patients first admitted before or after the reorganization of psychiatric care in Sweden.	Bülow P.H., et al., 2022 [41]	*International Journal of Mental Health Systems*	Pre-post-observational study.	Sweden restructured its psychiatric care through sectorization, i.e., different regions organized inpatient and outpatient care independently. This reform led to shorter hospital stays, a reduction in the number of hospital beds, and improved access to psychiatric care. Compared with the post-sectorization group, the patients of the pre-sectorization period demonstrated lower levels of functioning and had more unmet needs, even when controlling for diagnosis.
18	Psychiatric reform and counter-reform: An analysis of a socio-political and sanitary crisis at national and regional level.	De Oliveira Nunes M., et al., 2019 [42]	*Ciencia & Saude Coletiva*	Narrative review.	This article addresses the uncertain political situation in Brazil regarding mental health. While psychiatric counter-reform is underway, marked by the reemergence of asylums, a paradoxical counter-counter-reform has emerged in the state of Bahia. The authors call for advocacy to prevent human rights violations.
19	The Effectiveness of Mental Health Rehabilitation Services: A Systematic Review and Narrative Synthesis.	Dalton-Locke C., et al., 2021 [43]	*Frontiers in Psychiatry*	Systematic literature review.	A systematic review shows that most patients can be successfully discharged from long-stay psychiatric hospitals into community settings without clinical deterioration. A substantial proportion of these individuals transition to more independent living arrangements, although some continue to require long-term support accommodation. A longer stay in hospital is associated with less favorable outcomes.
20	The transition towards community-based mental health care in the European Union: Current realities and prospects.	Vandoni M., et al., 2024 [44]	*Health Policy*	Narrative review.	The transition to community-based services has been implemented differentially across the European Union. Mental hospitals remain the prominent model of care in most countries, with a few countries endorsing a balanced care model that combines hospital and community care. Better data reporting is key to advancing research, policies, and practices.
21	Finding Common Ground for Diverging Policies for Persons with Severe Mental Illness.	Solomon P., Petros R., 2020 [45]	*The Psychiatric Quarterly*	Narrative review.	Violent incidents involving individuals with mental illness are estimated to account for approximately 4% of all violence in the US, with family members most often being the targets. People with mental health illness are significantly more likely to harm themselves than to harm others. The authors suggest policies that balance risk management with risk opportunity by investing in voluntary treatment approaches that emphasize shared decision-making.
22	The relationship between deinstitutionalization and quality of care in longer-term psychiatric and social care facilities in Europe: A cross-sectional study.	Taylor Salisbury T., et al., 2022 [46]	*European Psychiatry*	Cross-sectional study.	Although deinstitutionalization is associated with higher quality of care, particularly in five key domains—promotion of self-management and autonomy, incorporation of recovery-based practice, availability of treatments and interventions, and better built and therapeutic environments—it has not been significantly linked to better quality of life or care experiences. Transition to community care shows improvements in treatment and outcomes, regardless of the country’s current level of deinstitutionalization.
23	Owning our madness: Contributions of Jamaican psychiatry to decolonizing Global Mental Health.	Hickling F.W., 2019 [47]	*Transcultural Psychiatry*	Narrative review.	Deinstitutionalization in Jamaica was influenced by the country’s history of colonization. Jamaica successfully implemented the gradual downsizing and dismantling of mental hospitals, alongside the establishment of community-based mental health care. The article highlights several successful interventions, including the integration of mental health into general medicine, the Diversion at the Point of Arrest Program, and the reduction in mental health stigma.
24	Evolution of forensic psychiatry in Italy over the past 40 years (1978–2018).	Ferracuti S., et al., 2019 [48]	*The International Journal of Law and Psychiatry*	Narrative review.	The infamous Mental Health Reform in 1978 (Law 180) in Italy failed to address the area of forensic psychiatry. Under the new legislation, inpatient forensic psychiatric hospitals have been replaced by community structures led by psychiatrists and supported by public security staff, with a focus on rehabilitation. The article illustrates the discrepancy between civic and penal law: civic law permits compulsory treatment only when there is a medical need, while penal law allows for security measures if there is a perceived risk of criminal recidivism. This misalignment can result in psychiatric treatment of individuals who may not require it and, in some cases, may even feign illness in order to receive certain benefits.
25	Caregiving burden in family caregivers of patients with schizophrenia: A qualitative study.	Tamizi Z., et al., 2020 [49]	*Journal of Education and Health Promotion*	Qualitative study.	Caring for a patient with schizophrenia places a significant burden on family caregivers. The burden is influenced by factors such as the duration of contact with the patient, the demands of long-term caregiving, the caregiver’s knowledge about the disease, and the degree of integration between the family and the medical team. Proposed future strategies include identifying high-risk caregivers; designing appropriate family-based intervention; and encouraging the active involvement of family members in shaping community care policies and services, such as post-discharge surveillance and home visits.
26	Sociodemographic, clinical and criminological characteristics of a sample of Italian Volterra REMS patients.	Lombardi V., et al., 2019 [50]	*International Journal of Law and Psychiatry*	Cross-sectional study.	Since the closure of forensic psychiatric hospitals in Italy, a new care structure for mental health patients with a history of criminal offenses has been established. This system is based on community residential units. The patient’s population is demographically similar to that of the former forensic psychiatric hospitals, with schizophrenia being the most common primary diagnosis and substance-related disorders the most frequent comorbidity. Due to the high turnover of patients, the authors anticipate future changes in certain sociodemographic, clinical, and criminological characteristics of this population.
27	A Comparison of Mental Health Care Systems in Northern and Southern Europe: A Service Mapping Study.	Sadeniemi M. et al., 2018 [51]	*International Journal of Environmental Research and Public Health*	Service mapping study.	The study compares deinstitutionalization between regions in Finland and Spain. A more structured community care service is associated with a lower suicide rate. In Finland, inpatient hospital days decreased by 54% between 1994 and 2015, accompanied by a 41% reduction in suicide rate. In Spain, the majority of patients with schizophrenia live with their families and are unemployed, whereas in Finland, only half as many patients live with their parents. The difference has contributed to a higher rate of transinstitutionalization into residential services in Finland.
28	Coercion in Outpatients under Community Treatment Orders: A Matched Comparison Study.	Nakhost A., et al., 2018 [52]	*The Canadian Journal of Psychiatry*	Matched comparison study.	Patients with serious mental illness who lack insight may be treated under mandated community treatment orders. Service users treated under such order reported significantly higher levels of perceived coercion compared with those in the control group. However, the perception of coercion was directly correlated with their previous experience with probation and inversely linked with the sense of procedural justice in their treatment.
29	Quality of care and its determinants in longer term mental health facilities across Europe; a cross-sectional analysis.	Killaspy H. et al., 2016 [53]	*Consortium Psychiatricum*	Cross-sectional analysis.	The Quality Indicator for Rehabilitative Care (QuIRC) is an international, standardized quality tool for the evaluation of long-term mental health facilities. In the ten European countries included in the study, the overall quality of care in units for long-term patients was found to be reasonable, although some units still require improvement. Higher quality scores were observed in smaller, community-based units; those serving patients with varying levels of disability; and mixed gender facilities.
30	The effects of national mental health plans on mental health services development in Chile: Retrospective interrupted time series analyses of national databases between 1990 and 2017.	Mundt A.P., et al., 2022 [19]	*International Journal of Mental Health Systems*	Retrospective, quasi-experimental, observational study.	Deinstitutionalization in Chile has been largely successful, with a significant reduction in both short- and long-stay psychiatric hospital beds, as well as a shift in funding away from psychiatric hospitals toward community care between 1990 and 2017. In recent years, however, there has been an increase in the number of psychiatric beds in general hospitals, forensic psychiatric hospitals, and short-stay units within psychiatric hospitals, despite the absence of formal policy supporting this trend. This effect may be attributed to the conversion of long- and medium-stay beds into acute and short stays within the same facility.
31	Community Engagement Mental Health Model for Home Treatment of Psychosis in Jamaica.	Nelson D., et al., 2020 [54]	*Psychiatric Services*	Narrative review.	Evidence suggests that Jamaican’s community-based treatment of patients with acute psychotic disorder is a viable strategy, demonstrating high levels of satisfaction among both service users and providers, as well as favorable clinical outcomes. Lower-income countries must adapt mental health care models developed in high-income countries to suit their own resources and contexts.
32	Challenges of Providing Home Care for a Family Member with Serious Chronic Mental Illness: A Qualitative Enquiry.	Mokwena K.E., Ngoveni A., 2020 [55]	*International Journal of Environmental Research and Public Health*	Qualitative study.	Following deinstitutionalization in South Africa, patients are sometimes discharged home to be cared for by their families, often with only medication and no support. Families have reported experiencing violence perpetrated by the patients, safety concerns, financial strain, emotional distress, and a desire for patients to remain in institutional care. Community-based outreach teams should extend their services to both patients and families, ensuring appropriate follow up and ongoing support.
33	An Innovative Approach to the Dismantlement of a Forensic Psychiatric Hospital in Italy: A Ten-year Impact Evaluation.	Leone L., et al., 2023 [56]	*Clinical Practice & Epidemiology in Mental Health*	Pre-post-study design.	The closure of forensic psychiatric hospitals in Italy and the subsequent opening of community care centers for forensic patients have led to improvements in patients’ mental health during the follow-up period, even among those with a history of long institutionalization and serious mental illness. The need for high control structures during the first two years post-discharge has decreased, and a high rate of workplace integration has been observed. There was a reduction in health care costs, and mental health professionals report the highest level of job satisfaction among health care workers in Italy regarding the quality of care they provide. On the other hand, the study showed high mortality rate from among the initial group of discharges (18.2% of the sample) with an average age of death of just 49 years.
34	The Quality of Care Provided by Outpatient Mental Health Services in Georgia.	Chkonia E., et al., 2021 [57]	*Consortium Psychiatricum*	Cross-sectional observational study.	The transition to community-based care in Georgia, supported by an increased budget, the introduction of new standards, and development of mobile teams, has led to improved medication supply, a more integrated biopsychosocial treatment approach, and training and employment of more mental health professionals. The study also identified the remaining challenges, such as disturbances in redistribution of funding and personnel.
35	Psychiatric Reform in Rio de Janeiro: The current situation and future perspectives.	Fagundes Júnior H.M., et al., 2016 [58]	*Ciencia & Saude Coletiva*	Narrative review.	Deinstitutionalization in Rio de Janeiro has led to a progressive reduction in the number of beds in psychiatric hospitals and an increased role for community-based facilities. While both forms of mental health care still coexist, hospitalization often disrupts continuity of care, leading to repeated admissions. Many individuals seek urgent or emergency psychiatric services. Remaining challenges include the integration of psychiatric beds in general hospitals; the deinstitutionalization of individuals with prolonged hospital stays; and the need for more residential facilities and expanded community services, particularly for people with substance use disorders.
36	The Revolving Door Phenomenon in the Romanian Mental Health System.	Păun R-M, et al., 2025 [59]	*Alpha Psychiatry*	Cross-sectional study.	Deinstitutionalization without sufficiently developed community care often leads to the “revolving door” phenomenon, characterized by at least three hospitalizations in a two-year period. In the Romanian mental health system, the most likely explanation for this pattern is the higher concentration of resources in inpatient units, leaving patients with low socioeconomic status without viable community-based alternatives for ongoing monitoring and treatment.
37	Forty years of the Law 180: The aspirations of a great reform, its successes and continuing need.	Mezzina R., 2018 [60]	*Epidemiology and Psychiatric Sciences*	Narrative review.	Italy implemented the most progressive mental health law, which resulted in the closure of psychiatric hospitals. The law was centered around patients’ needs and rights; however, 40 years later, some drawbacks can be seen. One of them is the lack of protections around involuntary treatment, as there is no consistent, time-limited review of such admissions by independent bodies, as recommended by the World Health Organization. There is a regional variation in the quality of care, with some areas failing to provide adequate crisis services and long-term support.
38	Shifting Trends in Admission Patterns of an Acute Inpatient Psychiatric Unit in the State of New York.	Shah B., et al., 2020 [61]	*The Cureus Journal of Medical Science*	Retrospective observational study.	In the State of New York, there has been a decline in the number of depressive disorder cases treated in emergency settings, which may be linked to improvements in community-based care. In contrast, admissions related to substance use disorders, personality disorders, trauma-related disorders, and neurodevelopment disorders have higher numbers of admissions, which can be explained by their common comorbidities and complex issues, including violence, that require inpatient care. A significant upward trend in the use of restraints has also been observed, which could be mitigated with additional de-escalation training and the implementation of larger units with single rooms to reduce aggression. A decrease in the length of stay has been noted, possibly attributable to both the improvement in treatment options and cost-cutting efforts, as well as the availability of effective community-based options.
39	Factors associated with the revolving door phenomenon in patients with schizophrenia: Results from an acute psychiatric hospital in Romania.	Dionisie V., et al., 2025 [62]	*Frontiers in Psychiatry*	Observational and retrospective cohort study.	The “revolving door” phenomenon, characterized by frequent hospital readmissions, emerged following deinstitutionalization in Romania. Risk factors influencing this phenomenon among patients with schizophrenia include male sex, younger age, comorbid substance or alcohol use disorders, and tendencies toward physical or verbal aggression.
40	Accommodation and Health Costs of Deinstitutionalized People with Mental Illness Living in Residential Services in Brazil.	Razzouk D., 2019 [63]	*PharmacoEconomics-Open*	Cross-sectional study.	Accommodation costs were not significantly influenced by patient profile variables; rather, the region and duration of the hospital stay or the stay in residential care were the main cost predictors. Some residents living more autonomously in independent housing occasionally returned to fully staffed homes. Both health and non-health community services were underutilized. Despite the expectations of deinstitutionalization, findings indicated that patients continued to experience a poor quality of life. The main contributing factors to this were symptom severity, limited social skills, reduced self-care abilities, and poor education.
41	An international comparison of the deinstitutionalization of mental health care: Development and findings of the Mental Health Services Deinstitutionalization Measure (MENDit).	Taylor Salisbury T., et al., 2016 [64]	*BMC Psychiatry*	Instrument development and validation study.	The MENDit is a tool used to assess a country’s level of deinstitutionalization. It evaluated factors such as the existence of specific mental health legislation, dedicated policies and budgets, and the integration of mental and physical health care systems, ideally structured around service users’ needs and focused on promoting autonomy. Inpatient psychiatric units continue to operate in all countries in Europe except Italy and Iceland, while 63% of countries report offering some degree of community residential care. Some authors argue that the rising numbers of psychiatric beds outside of traditional psychiatric hospitals reflect reinstitutionalization, but the article suggests that a truly deinstitutionalized system is the one that provides the most appropriate setting and level of support based on users’ needs. Furthermore, the number of psychiatric beds per capita does not strongly correlate with deinstitutionalization. One of the key challenges to effective deinstitutionalization remains the shortage of trained mental health professionals.
42	Observed Outcomes: An Approach to Calculate the Optimum Number of Psychiatric Beds.	O’Reilly R., et al., 2019 [65]	*Administration and Policy in Mental Health and Mental Health*	Literature review.	In most high-income countries, the numbers of psychiatric beds per capita has decreased. This shortage of available psychiatric beds is having serious consequences for the quality and safety of patient care. The authors argue that there is a threshold for the safe minimal number of psychiatric beds and examine the relationship between bed availability and key hospital performance indicators, as well as population-level patient outcomes, in order to estimate the minimum and optimal bed requirements. The identified factors include out-of-area placements, boarding, involuntary admission, bed occupancy rates, reduction in length of stay, acuity levels in inpatient units, readmission rates, homelessness, suicide rates, all-cause mortality, violent crime and incarceration rates, and caregiver burden. To accurately determine the threshold, comprehensive data on hospitalizations across countries is required.
43	How mental health service systems are organized may affect the rate of acute admissions to specialized care: Report from a natural experiment involving 5338 admissions.	Myklebust L.H., et al., 2017 [66]	*Sage Open Medicine*	Observational study.	Locally integrated services, including the availability of local psychiatric beds, may be more responsive to patients’ needs and can help reduce the need for acute admissions to specialized psychiatric services. Patient characteristics, such as diagnoses and the use of coercion measures, are important predictors of acute admission.
44	Investigating the influence of contextual factors in the coordination of chronic mental illness care in a district health system.	Mandlenkosi Phehlukwayo S., Mahlako Tsoka-Gwegweni J., 2018 [67]	*African Health Sciences*	Qualitative multiple case study.	The research in a region of South Africa indicates deinstitutionalization of patients with chronic mental health illness was poorly planned and accompanied by insufficient outpatient care. This has resulted in fragmented services, reduced quality of care, and decreased safety of staff.
45	From asylums to deinstitutionalization and after: An analytic review.	Balbuena Rivera F., 2024 [68]	*International Journal of Social Psychiatry*	Analytic review.	The dismantling of asylums and the introduction of community-based care—although partly driven by economic factors and overly optimistic expectations of pharmacological treatments—led to significant changes in the care of mental health patients, including improvements in institutional conditions. However, this shift has failed to adequately support patients with the most severe forms of chronic mental illness and their families. Residential units and hospitals serve essential functions that cannot be easily eliminated, as evidenced by current challenges in forensic care, incarceration rates, and acute inpatient admissions.
46	On a long, narrow road: The mental health law in Turkey.	Artvinli, F., Uslu, M.K.B., 2023 [69]	*International Journal of Law and Psychiatry*	Narrative review.	The current legal framework, namely, the Turkish Civil Code, lacks comprehensive framework for involuntary treatment or compulsory hospitalization. In comparison, involuntary hospitalization rates across European countries range from 3.2% to 21.6%, while studies indicate that in Turkey, up to 85% of psychiatric hospitalizations may be involuntary. The article highlights the urgent need for legislation that protects individuals from stigmatization, discrimination, social exclusion, and human rights violations.
47	The impact of the Mental Health Act revision for deinstitutionalization in Korea on the crime rate of people with schizophrenia.	Kim A.M., Sohn J.H., 2023 [70]	*Psychiatry Research*	Quasi-experimental study.	Following the enactment of the Mental Health and Welfare Act in Korea in 2016, a decrease in hospital admissions and the number of psychiatric beds was observed, along with a slight decline in involuntary admissions. Compared with the general population, patients with paranoid schizophrenia had lower rates of rape, violence, intellectual crimes, and public order offenses but a higher rate of robbery. In 2021, the rates of murder, attempted murder, and arson committed by this patient group were 5.3, 6.5, and 11.4 times higher, respectively, than those in the general population. After the implementation of the law, crime rates among individuals with paranoid schizophrenia initially increased and then decreased. No statistically significant effect on the murder rate was observed following deinstitutionalization; however, the findings suggest positive association between deinstitutionalization and crime rate, which may be attributed to underdeveloped community-based care services.

## Data Availability

No new data were created or analyzed in this study.
